# The effect of clinical features and glucocorticoids on biopsy findings in giant cell arteritis

**DOI:** 10.1186/s12891-016-1225-2

**Published:** 2016-08-24

**Authors:** Karin Jakobsson, Lennart Jacobsson, Aladdin J. Mohammad, Jan-Åke Nilsson, Kenneth Warrington, Eric L. Matteson, Carl Turesson

**Affiliations:** 1 Department of Clinical Sciences, Rheumatology, Lund University, Malmö, Sweden; 2 Department of Rheumatology, Skåne University Hospital, S-205 02 Malmö, Sweden; 3 Department of Rheumatology & Inflammation Research, The Sahlgrenska Academy, University of Gothenburg, Institute of Medicine, Gothenburg, Sweden; 4 Vasculitis and Lupus Clinic, Addenbrooke’s Hospital, Cambridge, UK; 5 Division of Rheumatology, Mayo Clinic College of Medicine, Rochester, MN USA

**Keywords:** Giant cell arteritis, Temporal artery biopsy, Histopathology findings, Glucocorticoid treatment

## Abstract

**Background:**

To investigate the effect of baseline clinical characteristics and glucocorticoid treatment on temporal artery biopsy (TAB) findings in patients with giant cell arteritis (GCA).

**Methods:**

Individuals who developed GCA after inclusion in two population-based health surveys were identified through linkage to the local and the national patient registers. In addition, other patients diagnosed with GCA at the Departments of Internal Medicine and Rheumatology at an area hospital were included. A structured review of medical records and TAB pathology reports was performed. The presence or absence of giant cells, granuloma, fragmented internal elastic lamina, fibrosis and grade of inflammatory infiltrates were recorded.

**Results:**

In 183 cases with a confirmed clinical diagnosis of GCA, 139 were biopsied after start of glucocorticoids (median treatment duration 3 days; interquartile range 2–5). Patients with a positive TAB (77 %) had significantly higher C-reactive protein (CRP; *p* = 0.007) and erythrocyte sedimentation rate (ESR; *p* = 0.03) at the time of clinical diagnosis. A positive TAB tended to more common in women, but there was no difference in the proportion of patients with polymyalgia rheumatica or visual symptoms.

Patients biopsied before or on the same day as initial treatment where more likely than those biopsied 1–3 days after treatment start to have positive biopsy [odds ratio (OR) 2.86; 95 % CI 1.06–7.70] as well as inflammatory infiltrates (OR 3.30; 95 % CI 1.15–9.49).

There was no significant difference in the proportions of a fragmented internal lamina (*p* = 0.86), giant cells (*p* = 0.10), granuloma (*p* = 0.19), minor inflammatory infiltrates (*p* = 0.47), major inflammatory infiltrates (*p* = 0.09), or overall positive biopsy (*p* = 0.17) report by treatment duration comparing: ≤ 0 days, 1–3 days, 4–6 days, 7–28 days. Among those biopsied 7–28 days after start of treatment, 80 % of TABs were positive, and histopathology features were not substantially different from those biopsied after shorter glucocorticoid treatment.

**Conclusion:**

Biopsies were more likely to be positive and have characteristic histopathologic features in patients with high CRP and ESR, and prior to start of corticosteroid treatment TABs taken 1–4 weeks after initiation of glucocorticoid treatment reveal changes consistent with GCA and therefore still yields clinically useful information for the diagnosis.

**Electronic supplementary material:**

The online version of this article (doi:10.1186/s12891-016-1225-2) contains supplementary material, which is available to authorized users.

## Background

Clinical findings as well as histopathologic evidence of vasculitis are used for the diagnosis of giant cell arteritis (GCA). Temporal artery biopsy (TAB) is often employed in diagnosing GCA and in excluding other conditions.

Previous studies have shown that higher C-reactive protein (CRP) [1], erythrocyte sedimentation rate (ESR) [1, 2], thrombocytosis [1, 3] and anemia [2] are predictive for a TAB result compatible with GCA. Furthermore, higher age [4, 5], ethnicity [5] as well as an abnormal temporal artery on physical examination and constitutional symptoms [3] have been found to be significant predictors for a positive TAB in some studies, although others have failed to demonstrate a significant correlation between age, ethnicity or ESR and findings on TAB [6].

The Initial Systemic Inflammation Reaction (ISIR) is a measure of inflammation scored by the presence of the following five parameters: fever, anemia, thrombocytosis, leucocytosis and ESR > 100 mm/h [7]. The finding on TAB of inflammatory infiltrates confined to the adventitia at diagnosis has been correlated with lower levels of ISIR and a tendency towards a faster rate of glucocorticoid discontinuation [7].

There are conflicting data on the effect of glucocorticoid treatment on TAB findings in patients with GCA. An early study on the subject showed that biopsy findings were partly masked after only one week of glucocorticoid treatment [8]. Other studies have on the contrary shown changes consistent with temporal arteritis in TAB specimens from patients biopsied two [6, 9] or four [4] weeks of glucocorticoid treatment. Finally, recent preliminary reports from a multicentre study have reported a rapid resolution of the temporal artery halo signs demonstrated on ultrasonography [10] and a wide variety of TAB findings [11] after short term (mainly ≤ 1 week) glucocorticoid treatment.

Font and co-workers concluded that, although some signs of inflammation remained after long-term treatment, glucocorticoid treatment caused changes in the histological features of TAB [12]. In all 35 GCA cases in their study, a mix of lymphocytes with mononuclear cells and epithelioid histiocytes located in the outer muscle layer and the adventitia, as well as loss of the internal elastic lamina were observed. The likelihood of finding diffuse infiltrates in the arterial wall and giant cells was lower if the patient had been on glucocorticoid treatment for more than 14 days.

The interpretation of TAB findings is a key issue for the management of GCA. The purpose of this study was to investigate histopathology features in TAB from a well-defined group of patients with a clinical diagnosis of GCA, and assess how these features relate to baseline inflammatory markers and timing of initiation of glucocorticoid treatment.

## Methods

### Patients

Incident cases of GCA among participants in two population-based surveys performed in the same catchment area as well as other patients with a diagnosis of GCA at a single centre, the University hospital, which provides all primary and secondary inpatient care in the area, were included in the present study. The two population-based health surveys were performed in Malmö, Sweden (current population 318,000; population during the screening period 1974–1996 between 229,000 and 247,000). Details on the Malmö Diet and Cancer Study (MDCS, *N* = 30447 subjects; 12121 men/18326 women) and the Malmö Preventive Medicine Project (MPMP, *N* = 33346; 22444 men/10902 women) have been described elsewhere [13–15]. Patients with a registered diagnosis of GCA after inclusion in the MDCS and through December 31, 2011 were identified in a study investigating predictors of GCA, as previously described [15]. Preliminary findings on TAB features in a subset of the included patients have been reported previously [16].

In addition to the incident cases in the MDCS and the MPMP, all other cases with a registered GCA diagnosis in the outpatient clinical administrative register between 1993 and 2008 at the Department of Rheumatology and between 1998 and 2008 at the Department of Internal Medicine at Malmö University Hospital were included in the study sample.

The medical records of the identified patients were subjected to a structured review. Inclusion in the present study required a validated diagnosis of GCA, based on available medical records and expert opinion. In addition, cases were classified according to the American College of Rheumatology (ACR) 1990criteria for the classification of GCA [17]. The 1990 ACR classification criteria were not mandatory for inclusion in this study. Some cases with typical clinical features but limited data regarding some parameters were included even though they didn’t fulfill the criteria.

### Data collection

The date of diagnosis was defined as the day when a tentative clinical diagnosis was made and glucocorticoid treatment was started. Clinical data regarding GCA extracted from clinical records included information corresponding to the ACR criteria, i.e. age, new headache, abnormality on physical examination of the temporal arteries, ESR and results of the TAB. In addition, the following data at the time of diagnosis were collected according to a structured protocol: CRP, haemoglobin, platelets count, smoking history, family history of rheumatic diseases, documented symptoms of polymyalgia rheumatica (PMR) at diagnosis, visual symptoms (defined as all reported new onset visual problems not explained by other factors than GCA) and permanent visual impairment, and the initial dose of glucocorticoids including initial intravenous treatment if given. Information on large vessel involvement and all vascular imaging studies performed up to the date of record review was also noted.

All cases with a confirmed clinical diagnosis of GCA, adequate TAB according to the pathologist and details available regarding dates of performing TAB and start of glucocorticoid treatment were included in this study. Of 228 incident cases, 183 fulfilled the criteria above and were included in the present investigation. The reasons for exclusion were: no adequate biopsy (*n* = 21); lack of information on date of start of glucocorticoids (*n* = 6); lack of information date of TAB (*n* = 31).

A structured review of TAB pathology reports for all identified cases was performed. The biopsies were mainly performed by otorhinolaryngologists who had access to a summary report from the referring physician. This report usually included a short medical history, current symptoms and information regarding treatment with glucocorticoids. The presence or absence of the following histopathology features was recorded: giant cells, granuloma, fragmented internal elastic lamina, inflammatory infiltrates and fibrosis. Major inflammatory infiltrates were defined as the presence of a large number of inflammatory cells in several layers of the vascular wall (adventitia/media/intima). In the absence of a detailed description of all layers, lesions described as “massive inflammation”, “typical GCA” or using similar wording were classified as major inflammatory infiltrates, whereas those described as “limited infiltrates”, “minor inflammation” etc. were classified as minor inflammatory infiltrates. All reports were reviewed by two of the authors (KJ and CT). The final judgment was based on consensus.

### Statistical analysis

Baseline parameters in TAB positive vs. TAB negative patients were compared using chi-square test, Student’s *T* test (for continuous parameters with a normal distribution) and the Mann-Whitney *U* test (for continuous parameters with a skewed distribution). The relationship between reported histopathology features and overall positive biopsy in those biopsied before start of treatment or on the same day, after 1–3 days, after 4–6 or after 7–28 days of glucocorticoid treatment was analysed using the chi-square test. Two cases with a very long duration (35 and 253 days) from the time of initiation of treatment to time of TAB were not included in the assessment of duration between initial treatment and TAB. Logistic regressions were performed analysing clinical characteristics of TAB positive vs. TAB negative patients as well as those with and without reported inflammation on pathology reports. In analyses by category of time on glucocorticoids before TAB, the group that had been treated for 1–3 days was used as the reference, as this may reflect standard of care with prompt initiation of treatment and an early TAB. The Mann-Whitney *U* test was used to assess differences in the distribution of category of time on treatment with glucocorticoids among those with vs. without specific histopathology features.

## Results

A total of 183 cases with confirmed GCA, a representative TAB and information available regarding the time between TAB and glucocorticoid treatment start were included (Table 1). 102 cases were recruited from the review of the participants in the population based health surveys, and 81 patients from the local clinical administrative registery.

**Table 1:** Characteristics of patients with giant cell arteritis undergoing temporal artery biopsy

Number of patients	183
Female sex	134 (73 %)
Age at GCA diagnosis (years) (mean)	74.3 (SD 8.97; range 49–95)
Positive biopsy	141 (77 %)
Fulfilled ACR criteria	175 (96 %)
Visual symptoms at diagnosis	91 (50 %)
Permanent visual loss	22 (12 %)
ESR at diagnosis (mm/h) (mean)	81 (SD 26.6)
Initial glucocorticoid dose (mg prednisolone)	Median 40 (IQR 40–60); Mean 51 (SD 37.3)
CRP at diagnosis (mg/l) (median)	99 (IQR 56–143)
Large vessel involvement during follow-up	22 (12 %)

*GCA* giant cell arteritis, *ACR* American College of Rheumatology, *ESR* erythrocyte sedimentation rate, *CRP* C-reactive protein, *SD* standard deviation, *IQR* interquartile range

The median time from start of glucocorticoid treatment to TAB was 3 days [interquartile range (IQR) 2–5; maximum 253 days]. No patient received other immunosuppressive drugs before the TAB. 44 patients (24 %) were biopsied either before glucocorticoids were initiated or on the same day. For these, the median time from TAB to treatment start was 2 days [IQR 0–3.75; maximum 22 days]. Patients with a positive biopsy (141 of 183, 77 %) had significantly higher CRP (median 101 mg/l; IQR 70–145 vs. median 70, IQR 28–119; *p* = 0.007) and ESR (mean 83.1 mm/1 hr, SD 27.1 vs. mean 72.6, SD 23.2; *p* = 0.03) at the time of clinical diagnosis (Table 2).

**Table 2:** Baseline features at time of diagnosis^a^ in patients with GCA with positive vs. negative biopsy

Feature	Biopsy positive (*n* = 141)	Biopsy negative (*n* = 42)	*p*
Age at GCA diagnosis (years)	74.3 (SD 8.8; range 49–95)	74.4 (SD 9.5; range 54–93)	0.94
Female sex	108 (77 %)	26 (62 %)	0.06
ESR mm/h; mean (SD)^b^	83.1 (27.1)	72.6 (23.2)	0.03
CRP mg/l; median (IQR)^b^	101 (70–145)	70 (28–119)	0.007
Time from glucocorticoid treatment to TAB, days (median; IQR)	2 (0–4)	2 (1–5)	0.62
Initial steroid dose, mg (median; IQR)^b^	40 (40–60)	50 (40–60)	0.18
Symptoms of PMR at diagnosis	38 (27 %)	15 (36 %)	0.27
Visual symptoms at diagnosis	68 (48 %)	23 (55 %)	0.46
Permanent visual impairment	20 (14 %)	2 (5 %)	0.10
Current smoking^b^	73 (52 %)	25 (60 %)	0.43
Ever smoking^b^	103 (73 %)	32 (76 %)	0.83
Large vessel involvement during the follow-up	19 (13 %)	3 (7 %)	0.27

*ESR* erythrocyte sedimentation rate, *CRP* C-reactive protein, *PMR* polymyalgia rheumatica, *SD* standard deviation, *IQR* interquartile range

^a^Defined as the day when a tentative clinical diagnosis was made and glucocorticoid treatment was started

^b^Available data from 171 patients on ESR, 136 patients on CRP, prednisolone dose available from 177 patients, current smoking at diagnosis from 98 patients and from 135 patients on ever smoking

### Comparison of those who underwent TAB before or after starting glucocorticoid therapy

There was a greater proportion of women among those who were biopsied before or on the same day as initiation of glucocorticoid treatment, compared to those biopsied after treatment start (*p* = 0.02). The subset with a pre-treatment biopsy also had higher ESR (median 88 vs. 78; *p* = 0.04), but not CRP, levels at baseline. They were also less likely to have visual impairment at diagnosis (36 % vs. 54 %; *p* = 0.04), and they were treated with a lower dosage of oral glucocorticoids (median 40 mg/day; IQR 40–50 vs. median 50 mg/day; IQR 40–60; *p* = 0.005).

### Timing of TAB in relation to histopathology findings

Two cases with a very long duration (35 and 253 days) from the time of initiation of treatment to time of TAB were not included in the assessment of duration between initial treatment and TAB. Both these patients had a positive TAB and fulfilled the ACR criteria for GCA. TAB revealed inflammatory infiltrates and fragmented internal elastic lamina in both, and one had giant cells.

We compared pathology report findings in patients who started treatment with glucocorticoids after TAB or on the same day with those who had been treated for 1–3 days, 4–6 days and 7–28 days (Table 3). Overall, the interobserver agreement in the pathology report review was 93 %, ranging from 100 % (for the presence/absence of granuloma) to 78 % (for inflammatory infiltrates: major/minor/none) for the different features studied.

**Table 3:** Features recorded in pathology reports, stratified by time from initiation of glucocorticoid treatment to biopsy

Time from treatment start to biopsy	≤0 days	1-3 days	4-6 days	7-28 days	*p*	*p* for trend *
	(*n* = 44)	(*n* = 74)	(*n* = 43)	(*n* = 20)		
Inflammatory infiltrates	39 (89 %)	52 (70 %)	35 (81 %)	15 (75 %)	0.12	0.95
Biopsy positive	38 (86 %)	51 (69 %)	34 (79 %)	16 (80 %)	0.17	0.64
Fragmented internal elastic lamina	20 (45 %)	33 (45 %)	21 (49 %)	10 (50 %)	0.86	0.65
Giant cells	21 (48 %)	23 (31 %)	23 (53 %)	8 (40 %)	0.10	0.73
Granuloma	9 (20 %)	6 (8 %)	4 (9 %)	3 (15 %)	0.19	0.28
Fibrosis	4 (9 %)	15 (20 %)	8 (19 %)	4 (20 %)	0.47	0.24
Minor inflammatory infiltrates ^a^	13 (30 %)	27 (36 %)	14 (33 %)	6 (30 %)	0.87	0.94
Major inflammatory infiltrates ^b^	23 (52 %)	23 (31 %)	21 (49 %)	9 (45 %)	0.09	0.90

^a^Lesions described as “limited infiltrates”, “minor inflammation” etc. were classified as minor inflammatory infiltrates

^b^Lesions described as “massive inflammation”, “typical GCA” or using similar wording were classified as major inflammatory infiltrates

^*^The Mann–Whitney *U* test was used to assess differences in the distribution of category of time on treatment with glucocorticoids among those with vs. without specific histopathology features

Patients who had a biopsy before treatment was initiated were more likely to have inflammatory infiltrates than those biopsied at a later time point. There was no significant difference in the distribution of proportions of positive TAB results between the four groups (*p* = 0.17). The lowest probability of finding inflammatory infiltrates overall, major inflammatory infiltrates and giant cells was in those treated for 1–3 days prior to biopsy. Patients in this group were also the most likely to have been treated with intravenous glucocorticoids prior to biopsy (12 individuals (16 %) vs. 3 (7 %) and 2 (10 %) in the groups with total pre-biopsy glucocorticoid treatment of 4–6 and 7–28 days, respectively). The dose of intravenous glucocorticoids was usually 500 mg methylprednisolone per day, given for 1–3 days.

There were no significant differences in the proportions with a fragmented internal elastic lamina, giant cells, granuloma, or overall positive biopsy report by treatment duration (Table 3). There were no differences in the distribution of category of time on treatment with glucocorticoids for the presence of any of the histopathology features investigated, including major or minor inflammatory infiltrates (Table 3).

### Predictors for positive findings in TAB

By bivariate logistic regression, TAB performed before or on the same day as initial treatment was associated with histologic findings consistent with GCA (OR 2.86; 95 % CI 1.06–7.70) compared to those biopsied 1–3 days after treatment initiation, although this association did not reach statistical significance in analysis adjusted for sex (OR 2.54; 95 % CI 0.93–6.96). Women tended to be more likely to have a positive biopsy (OR 2.01; 95 % CI 0.97–4.20). This pattern was less marked in analysis adjusted for category of time on glucocorticoids (OR 1.73; 95 % CI 0.81–3.68). There were no differences in age at diagnosis or number of days from glucocorticoid treatment to TAB in analyses comparing patients with positive vs. negative biopsy. Neither current nor ever smoking were predictive of a positive biopsy (Table 4).

**Table 4:** Potential predictors at time of diagnosis^a^ for positive temporal artery biopsy in patients with GCA

Feature	Odds ratio	95 % CI	*p*
Sex (female vs male)	2.01	0.97–4.20	0.06
Age (per year)	1.00	0.96–1.04	0.94
Time from glucocorticoid treatment to TAB (per day)	1.02	0.95–1.09	0.65
Current smoking vs non-current smoking^b^	0.74	0.35–1.56	0.43
Ever smoking vs never smoking^b^	0.90	0.36–2.28	0.83
ESR (per mm/h)	1.02	1.00–1.03	0.04
CRP (per mg/l)	1.01	1.00-1.02	0.01
Time from glucocorticoid treatment to TAB			
< 1 day^c^	2.86	1.06–7.70	0.04
1–3 days	1.00 (reference)		
4–6 days	1.70	0.70–4.13	0.24
7–28 days	1.80	0.54–6.00	0.34
ESR (mm/h) ^*^^b^			
Quartile 1 (20–61)	1.00 (reference)		
Quartile 2 (62–79)	0.45	0.17-1.20	0.11
Quartile 3 (80–99)	1.14	0.43-3.01	0.80
Quartile 4 (100–150)	4.27	1.09-16.82	0.04
CRP (mg/l) ^**^^b^			
Quartile 1 (4–56)	1.00 (reference)		
Quartile 2 (57–98)	2.89	0.94-8.86	0.06
Quartile 3 (99–142)	4.64	1.33-16.23	0.02
Quartile 4 (143–478)	2.39	0.81-7.04	0.12

*TAB* temporal artery biopsy, *ESR* erythrocyte sedimentation rate, *CRP* C-reactive protein, *CI* confidence interval

^*^ *p* for trend 0.01

^**^ *p* for trend 0.07

^a^Defined as the day when a tentative clinical diagnosis was made and glucocorticoid treatment was started

^b^Available data: ESR from 171 patients; CRP 136 patients; current smoking at diagnosis 98 patients; ever smoking 135 patients.

^c^Includes individuals biopsied before start of glucocorticoid treatment

Patients in the highest quartile of ESR (100–150 mm/1 h) were four times more likely to have a positive biopsy (OR 4.27; 95 % CI 1.09–16.82), as were patients with CRP levels between 99 and 143 mg/l (third quartile) (OR 4.64; 95 % CI 1.33–16.23), compared to those in the lowest quartile of ESR and CRP, respectively. There was a progressively increasing chance of a positive biopsy with higher quartiles of ESR (*p* for trend: 0.01), and a similar tendency for CRP (*p* for trend: 0.07). The distribution of males and females was similar in quartiles of both ESR and CRP (data not shown).

Female sex tended to be a predictor for the presence of inflammatory infiltrates in pathology report (Table 5). Biopsy specimens from patients who underwent TAB before start of glucocorticoids, or on the same day as treatment initiation were significantly more likely to have inflammatory infiltrates (OR 3.30; 95 % CI 1.15-9.49) compared to those biopsied after 1–3 days of treatment. High CRP (third or fourth quartile, i.e. ≥ 99 mg/l) was also predictive of inflammatory infiltrates in the TAB, but there was no such association with ESR (Table 5). Higher quartiles of ESR and CRP were significantly associated with a progressively greater likelihood of presence of inflammatory infiltrates (*p* = 0.03 and *p* = 0.01, respectively).

**Table 5:** Potential predictors at time of diagnosis for inflammatory infiltrates recorded in pathology reports

Feature	Odds ratio	95 % CI	*p*
Sex (female vs male)	1.93	0.91–4.06	0.09
Age (per year)	1.01	0.97–1.05	0.61
Time from glucocorticoid treatment to TAB (per day)	1.01	0.96–1.07	0.66
Current smoking vs non-current smoking^c^	0.65	0.30–1.41	0.27
Ever smoking vs never smoking^c^	0.62	0.22–1.75	0.37
ESR (per mm/h)	1.01	1.00–1.03	0.05
CRP (per mg/l)	1.01	1.00–1.02	0.01
Time from glucocorticoid treatment to TAB			
< 1 day^d^	3.30	1.15–9.49	0.03
1-3 days	1.00 (reference)		
4-6 days	1.90	0.74–4.62	0.19
7-28 days	1.27	0.41–3.92	0.68
ESR (mm/h) ^a,c^			
Quartile 1 (20–61)	1.00 (reference)		
Quartile 2 (62–79)	0.51	0.19–1.36	0.18
Quartile 3 (80–99)	1.14	0.43–3.01	0.80
Quartile 4 (100–150)	3.13	0.90–10.90	0.07
CRP (mg/l) ^b,c^			
Quartile 1 (4–56)	1.00 (reference)		
Quartile 2 (57–98)	2.89	0.94–8.86	0.06
Quartile 3 (99–142)	4.64	1.33–16.23	0.02
Quartile 4 (143–478)	3.60	1.11–11.62	0.03

*TAB* temporal artery biopsy, *ESR* erythrocyte sedimentation rate, *CRP* C-reactive protein, *CI* confidence interval

^a^ p for trend 0.03

^b^ p for trend 0.01

^c^Available data: ESR from 171 patients; CRP 136 patients; current smoking at diagnosis 98 patients; ever smoking 135 patients

^d^Includes individuals biopsied before start of glucocorticoid treatment

In logistic regression models using the category of patients biopsied before start of glucocorticoids as reference, there was no linear progression of the odds ratio for positive biopsy (Additional file 1: Table S1) or for inflammatory infiltrates (Additional file 1: Table S2).

## Discussion

In this study of a population-based sample of patients with a clinical diagnosis of GCA, we found no significant change in the TAB results with longer time on glucocorticoids. Importantly, histopathology features in those biopsied 7–28 days after start of treatment were not substantially different from those biopsied after shorter glucocorticoid treatment. Patients who were biopsied before or on the same day as initial treatment were more likely than those biopsied shortly (1–3 days) after initiation of treatment to have a positive biopsy, as well as inflammatory infiltrates on TAB. The group treated for 1–3 days with glucocorticoids had the lowest probability for a positive biopsy report, presence of major inflammatory infiltrates and giant cells. Possibly, early high glucocorticoid exposure could contribute to this pattern. This includes intravenous treatment, which was slightly more frequent among those biopsied after 1–3 days. However, Font et al. compared 6 patients with intravenous treatment to 29 who received oral glucocorticoids only, and did not find any differences in histopathology findings [12]. The mean oral starting dose of glucocorticoids in that study (74 mg/day) was substantially higher than the average starting dose given in this study (51 mg/day) [12]. Unfortunately, data on the tapering of glucocorticoids are not available from the present study.

Women tended to be more likely to have a positive biopsy, but neither current nor ever smoking was predictive of biopsy findings. Higher quartiles of ESR and CRP were predictive for positive biopsy, and in the case of CRP also for the presence of inflammatory infiltrates. We did not find any association between age, initial glucocorticoid dose, symptoms of PMR or visual symptoms at time of diagnosis and biopsy positivity. Cases biopsied before or on the same day as initiation of glucocorticoids were more often female, had significantly higher ESR and were less likely to have visual impairment at diagnosis.

The reason that women were more likely to undergo TAB before or on the same day as initial treatment is unclear. It may be that because GCA is more common in women, the diagnosis may be suspected earlier than in men. This observation could also reflect differences in presentation between male and female patients with GCA. This should be further explored.

In the present study, as many as 50 % of the patients had visual symptoms at the time of diagnosis. This figure is higher than that reported in most previous studies [18, 19]. On the other hand, the proportion of patients with permanent visual impairment was slightly lower compared to what has been reported previously [19]. This likely reflects that most of the patients in the present study received adequate treatment with glucocorticoids as soon as the diagnosis was suspected, in particular when visual symptoms were present.

Font et al. [12] found that patients with GCA who had been treated with glucocorticoids for more than 14 days before the TAB had inflammatory infiltrates that were limited to the adventitia-media junction, whereas those with shorter treatment were more likely to have diffuse infiltrates. By contrast, we found that major inflammatory infiltrates did not seem to respond rapidly to treatment. Neither could we confirm their findings suggesting that giant cells vanished after even a few days of treatment, as the proportion with documented giant cells in the TAB in our study was similar among those biopsied after 7–28 days of glucocorticoid treatment compared to those with an earlier TAB. These discrepancies may be explained partly by differences in glucocorticoid dose (see above), and also by methodological differences, in particular case ascertainment. Font et al. defined positive biopsy by inflammatory infiltrates containing both lymphocytes and epithelioid histiocytes, whereas in our cases the histopathology was judged consistent with GCA based on the overall impression of the clinical pathologist. Furthermore, geographic differences and differences in sample size (183 patients in the present study compared to 35 in the study by Font et al.) may contribute to the difference in findings.

Our results suggest that glucocorticoid treatment do not significantly affect the features of inflammation seen in TAB the first weeks of treatment. Deng et al. have suggested that some specific parts of the inflammation might persist during treatment while other parts might respond more rapidly, reporting that glucocorticoids down-regulate IL-17 production by Th17 cells, while Th1 cells producing interferon-γ- persist despite long term treatment [20]. This is a hypothesis that cannot be confirmed or rejected by the present study, and should be further investigated.

A limitation of this study is that information regarding TAB histopathology was collected from pathology reports only. Another limitation of this study is that the pathology reports were collected over an extended period, 1974–2011. The pathologists’ interpretation and description of the TAB reports may have changed over time. Strengths of the study include the population-based approach, reflecting that the patients are representative for GCA cases managed in the area, and the availability of complete medical information and histopathology data.

## Conclusions

Biopsies were more likely to be positive and have characteristic histopathologic features in GCA in patients with high CRP and ESR, and when the biopsy was performed prior to start of glucocorticoid treatment. TABs taken 1–4 weeks after starting glucocorticoids still reveal changes consistent with GCA, including inflammatory infiltrates and/or giant cells, in a majority of patients with a clinically suspected diagnosis. Delayed TAB still yields clinically useful information for the diagnosis of GCA.

## Additional file

Additional file 1: Table S1. Potential predictors at time of diagnosis* for positive temporal artery biopsy in patients with GCA. **Table S2.** Potential predictors at time of diagnosis for inflammatory infiltrates recorded in pathology reports. (DOC 34 kb)

## Abbreviations

ACR: American College of Rheumatology
CRP: C-reactive protein
ESR: Erythrocyte sedimentation rate
GCA: Giant cell arteritis
ISIR: Initial systemic inflammation reaction
MDCS: Malmö Diet and Cancer Study
MPMP: Malmö Preventive Medicine Project
PMR: Polymyalgia rheumatic
TAB: Temporal artery biopsy
