# Supplementary figures and images for: The effect of clinical features and glucocorticoids on biopsy findings in giant cell arteritis
Source: BMC Musculoskelet Disord. 2016 Aug 24;17(1):363. doi: 10.1186/s12891-016-1225-2 (PMC4997683; doi:10.1186/s12891-016-1225-2)

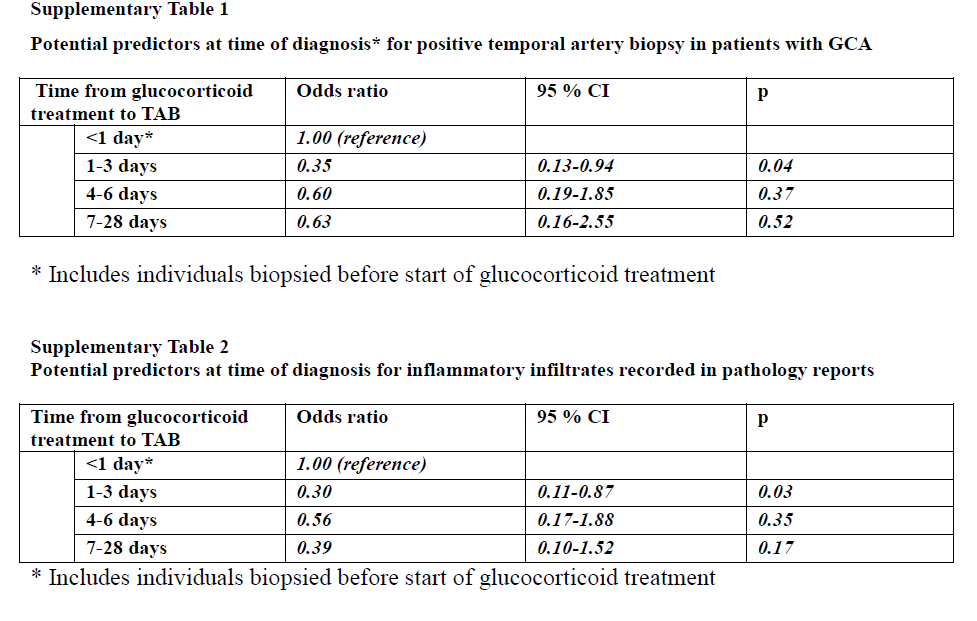

Supplement: Additional file 1: Table S1. — Potential predictors at time of diagnosis* for positive temporal artery biopsy in patients with GCA. Table S2. Potential predictors at time of diagnosis for inflammatory infiltrates recorded in pathology reports. (DOC 34 kb) [file 12891_2016_1225_MOESM1_ESM.doc]
